# Development of an mHealth platform for HIV Care: Gathering User Perspectives Through Co-Design Workshops and Interviews

**DOI:** 10.2196/mhealth.9856

**Published:** 2018-10-19

**Authors:** Benjamin Marent, Flis Henwood, Mary Darking, EmERGE Consortium

**Affiliations:** 1 School of Applied Social Science University of Brighton Falmer United Kingdom

**Keywords:** mHealth, mobile applications, telemedicine, information technology, sexual health, HIV, self-management, patient participation, sociology, medical, community-based participatory research, health services

## Abstract

**Background:**

Despite advances in testing and treatment, HIV incidence rates within European countries are at best stable or else increasing. mHealth technology has been advocated to increase quality and cost-effectiveness of health services while dealing with growing patient numbers. However, studies suggested that mHealth apps are rarely adopted and often considered to be of low quality by users. Only a few studies (conducted in the United States) have involved people living with HIV (PLWH) in the design of mHealth.

**Objective:**

The goal of this study was to facilitate a co-design process among PLWH and clinicians across 5 clinical sites in the European Union to inform the development of an mHealth platform to be integrated into clinical care pathways. We aimed to (1) elicit experiences of living with HIV and of working in HIV care, (2) identify mHealth functionalities that are considered useful for HIV care, and (3) identify potential benefits as well as concerns about mHealth.

**Methods:**

Between January and June 2016, 14 co-design workshops and 22 semistructured interviews were conducted, involving 97 PLWH and 63 clinicians. Data were analyzed thematically and iteratively, drawing on grounded theory techniques.

**Results:**

Findings were established into 3 thematic clusters: (1) *approaching the mHealth platform*, (2) *imagining the mHealth platform*, and (3) *anticipating the mHealth platform’s implications*. Co-design participants *approached* the mHealth platform with pre-existing concerns arising from their experiences of receiving or providing care. PLWH particularly addressed issues of stigma and questioned how mHealth could enable them to manage their HIV. Clinicians problematized the compatibility of mHealth with existing information technology systems and questioned which patients should be targeted by mHealth. *Imagining* the potential of mHealth for HIV care, co-design participants suggested *medical functionalities* (accessing test results, managing medicines and appointments, and digital communication channels), *social functionalities* (peer support network, international travel, etc), and *general features* (security and privacy, credibility, language, etc). Co-design participants also *anticipated* potential implications of mHealth for self-management and the provision of care.

**Conclusions:**

Our approach to co-design enabled us to facilitate early engagement in the mHealth platform, enabling patient and clinician feedback to become embedded in the development process at a preprototype phase. Although the technologies in question were not yet present, understanding how users approach, imagine, and anticipate technology formed an important source of knowledge and proved highly significant within the technology design and development process.

## Introduction

Since the availability of effective antiretroviral therapy (ART) by the end of the 1990s, HIV has transformed, in developed countries at least, from a fatal to a chronic disease. People living with HIV (PLWH) who have access to testing, treatment, and care can enjoy a good quality of life and the same life expectancy as the general population [1]. However, despite several advances in HIV testing and new biomedical HIV prevention modalities (such as early ART for prevention), the incidence rates within European countries are at best stable, and in some cases, they are even increasing [2]. In association with longer life expectancy, this leads to a continuously increasing number of people requiring long-term treatment and follow-up.

mHealth technologies, based on smartphone and Web 2.0 apps, are seen by policy makers, developers, and some medical professionals as an opportunity to increase the quality and cost-effectiveness of health services while dealing with growing numbers of patients [3-6]. Systematic reviews in the field of HIV care have highlighted that mHealth interventions have significant potential to support patients’ self-management and treatment adherence [7,8]. Self-management is understood as care that is led, owned, and undertaken by patients’ themselves. To this end, mHealth tools can provide patients with ubiquitous access to health data, information, and counseling beyond the face-to-face clinical encounter, which might reduce the need for routine clinical appointments and thus lower both the impact of HIV on patients’ lives and health care expenditure. Effective ART requires a 95% adherence to the antiretroviral medication regime. In this respect, mHealth can be utilized to send medication and appointment reminders to patients or provide them with information about prescribed medicines and drug interactions. A high level of treatment adherence contributes to viral suppression and thus increases HIV patients’ life expectancy and quality while decreasing the risk of forward transmission of HIV [9].

Systematic reviews identify several smartphone apps for HIV self-management and medication adherence available in app stores [10-12]. However, the authors highlight that most of these apps were infrequently downloaded and were considered of low quality by users as they did not have desirable features. The reviews stress the need for formative evaluations that include end users within the design, development, and implementation of mHealth devices to make them more accessible and meaningful. In addition, studies on the general adoption of health apps show that despite a vast range of available apps, only a small number are actually used [13]. If the ambitions of mHealth to improve quality and utilization of health care are to be realized, *co-design* processes that bring together health care providers, researchers, technology developers, and end users are crucial to produce useful and usable mHealth technologies.

Only a few studies have involved PLWH to inform the design of smartphone apps for HIV prevention, treatment, and care [14-16] or to test prototypes [17]. Furthermore, in the context of HIV care, clinicians are almost never considered as potential adopters of mHealth technology and so are rarely included in co-design initiatives. So far, only 1 study has assessed HIV clinicians’ attitudes toward mHealth and, thereby, outlined perspectives of how this technology could be integrated into clinical care pathways [18]. Moreover, the involvement of users—most often PLWH—tends to be restricted to the initial mHealth design phase, and only very recently are studies beginning to extend user involvement (focusing on young men who have sex with men) to the implementation phase of mHealth interventions [17,19]. Although critical decisions about what desired functionalities are included in the final product can be made in the design phase, it is only later in the implementation phase that actual experiences with mHealth devices can be captured and reflected upon. Finally, most participatory studies were conducted in the United States [14-19], and to our knowledge, there is no evidence of how PLWH and clinicians in European health care systems evaluate the potentials and risks of mHealth for use in HIV treatment and care.

In this paper, we address these gaps by presenting results from the first phase of an mHealth co-design process involving 97 PLWH and 63 clinicians from 5 clinical sites in the European Union (EU). We asked co-design participants to reflect, in the design phase, on the potentiality and risks they associated with mHealth in the context of HIV treatment and care and on the precise functionalities that they thought could support self-management of HIV. These findings were used to inform the development of a comprehensive mHealth platform that is currently being implemented within clinical treatment pathways in the 5 clinical sites. Later phases of the co-design work will seek to capture patients’ and clinicians’ experiences in the use of the platform to support continuous improvement as new pathways and technologies become embedded into HIV care.

## Methods

### The Evaluating mHealth Technology in HIV to Improve Empowerment and Health Care Utilization: Research and Innovation to Generate Evidence for Personalized Care Project

The co-design process presented in this paper is part of the *Evaluating mHealth Technology in HIV to Improve Empowerment and Health Care Utilization: Research and Innovation to Generate Evidence for Personalized Care (EmERGE) project* (please refer to [20]), which is funded under the EU’s Horizon 2020 Programme (project period: 2015-2020). The project aims to develop, implement, and evaluate an mHealth platform to support self-management among HIV patients in 5 clinical sites (Brighton, Antwerp, Zagreb, Barcelona, and Lisbon). The platform is currently being integrated into clinical HIV pathways and provides users (PLWH and clinicians) with smartphone and Web apps to facilitate access to personal health data and improve patient-provider communication. Through these functionalities, the EmERGE mHealth platform aims to reduce some routine clinic visits of HIV patients and support patients to better self-manage their own care. According to international guidelines, PLWH are currently seen every 3 to 6 months [21]. However, the EmERGE mHeaIth care pathway requires HIV patients to see their consultant face-to-face only every 12 months, while they can continuously monitor their blood results and maintain contact with their clinic through the smartphone app. Previous studies that have investigated the potential of mHealth in HIV care have suggested that such reductions in hospital visits are desirable for increasing the quality of life of PLWH [18]. The EmERGE project, as a whole, aims to validate the acceptability, usability, and effectiveness of the mHealth platform; assess its impact on patient self‐management and empowerment; analyze its cost-effectiveness; and disseminate the mHealth platform across various European health care settings as a sustainable, effective, safe, and economic modality for HIV care. The co-design process, outlined in this paper, constitutes an essential part of the *sociotechnical evaluation* work package, which seeks to identify and support factors that can help facilitate the successful introduction of the new care pathway. In the first year of the project (June 2015 to June 2016), in the platform’s initial *design phase*, we undertook a co-design process among potential users of the EmERGE platform—PLWH and clinicians. The results from this process are presented in this paper and have informed the technology development. Further research is currently being carried out, as the platform is implemented, to investigate how it reconfigures practices of HIV care [22]. Study approval was obtained from the ethics committee of the University of Brighton, the National Health Service (NHS) Health Research Authority, and governance boards at each clinical site.

### Facilitating Co-Design

Co-design research is recognized as an important means to establish the effective and responsible delivery of mHealth technologies [23-25]. In this section, we highlight how we engaged PLWH and clinicians in co-design to elicit current challenges of HIV care and to consider the potential uses and implications of integrating an mHealth platform within existing care pathways.

To initiate co-design, we worked closely with HIV clinicians and patient organizations, in particular, the European Aids Treatment Group (EATG) and its local partners within each study site. Collaboratively, we designed a protocol for engaging PLWH and clinicians alongside the iterative phases of the platform’s design and implementation. We decided to use co-design workshops as the main method through which potential users in design activities to develop ideas and identify challenges could be involved [26]. However, co-design workshops are also recognized as a challenging method. Time-constrained clinicians as well as some of the more vulnerable and often stigmatized HIV patients can find it difficult to participate in lengthy, group-based workshops. To attend to this potential shortcoming, individual interviews were offered as an alternative means to engage participants who were unwilling or unable to attend workshops.

With our clinical and PLWH-community partners, we established a schedule for workshops and interviews. First, the idea of using an mHealth platform for HIV care was presented as a narrative stimulus to identify ideas and new ways of *doing* HIV care. Thereafter, co-design activities were conducted in 3 phases that all drew on the *use situation* as a fundamental starting point for design [27]. First, participants were encouraged to discuss current practices of HIV care and uses of apps and mHealth technologies in their everyday lives. Second, they were prompted to imagine potential functionalities and features that could be provided through mHealth platforms and used within HIV care. Finally, participants were encouraged to anticipate potential implications of the use of mHealth platforms. Together, these open areas of questioning facilitated a range of views and experiences, including apparently contradictory ones.

PLWH-community partners at each of the study sites were trained as peer researchers to mobilize interest in the study, support the recruitment of patients, and facilitate co-design workshops and interviews in the local languages in cases where participants were not fluent in English. Community partners were regarded as trustworthy and knowledgeable by most PLWH, and they were experienced in moderating groups and working with vulnerable people.

### Recruitment and Data Collection

We attempted purposive sampling to recruit a diversity of PLWH (eg, gender, age, and nationality) and clinicians (eg, having a good representation of doctors and nurses) while accepting that fieldwork pragmatics (such as study timelines, access to potential participants, etc) will limit its success. A total of 97 PLWH and 63 clinicians were recruited into the co-design process. From the 97 PLWH we recruited, 19 were women. Moreover, 65 PLWH identified themselves as gay or lesbian, 26 as heterosexual, 3 as bisexual, and 3 belonging to none of these categories. The age range was 23 to 78 years with 11 participants under 30 years, 75 between 30 and 59 years, and 7 were 60 years or above (4 missing values). The length of diagnosis of HIV ranged from 0.5 to 31 years. Furthermore, 64 of the PLWH were working or studying, 21 were unemployed, and 12 were retired. PLWH had 14 different nationalities and 13 identified themselves as belonging to a migrant community. From the group of 63 clinicians, 19 were male. Clinicians included 40 doctors, 10 nurses, 4 psychologists, 4 pharmacists, 2 social workers, 2 nutritionists, and 1 sexologist.

A total of 14 workshops and 22 interviews were conducted at the offices of community partners, at hotels, or in the clinic, depending on what was appropriate. Table 1 shows the distribution of data collection and participants across the 5 study sites. The small number of clinicians (n=4) who participated in Zagreb is due to the size of the HIV clinic there (just 8 clinicians and a total of 862 patients). The other sites have between 2246 and 4846 patients and employ between 26 and 30 clinicians (according to 2014 data). In workshops indicated as *mixed*, PLWH and clinicians were involved in discussions together. While workshops with clinicians and *mixed* ones were conducted in English by the academic researcher, workshops with PLWH were facilitated in the local language by a community partner, with the academic researcher and a member of the EATG also present. An instant translator enabled the researchers to take notes and raise additional questions, as appropriate. Moreover, 15 interviews were conducted in English by the academic researcher and 7 interviews with PLWH by community partners in local languages. Throughout the data collection, the EATG member and the lead author exchanged and discussed their notes and debriefed impressions of the workshops with community partners. Workshops and interviews were audio recorded (average recording length: 95 min and 45 min, respectively). All recordings were transcribed verbatim and non-English transcripts were thoroughly translated. Participants provided written informed consent before the interview or workshop, and PLWH received €25 or £20 remuneration for participation.

### Analytic Approach

Data were analyzed thematically and iteratively, drawing on grounded theory techniques [28]. First, transcripts were carefully read-through by the first author of this paper (BM), and initial thoughts were documented. Open coding, conducted by BM, involved a sequential analysis where sentences with significant meaning were assigned first conceptual labels. These initial codes were then discussed among the whole academic research team (BM, FH, and MD) on a bimonthly basis, whereby they were continuously revised and established into thematic clusters and categories. Furthermore, preliminary findings were continuously discussed with the EATG, PLWH-community partners, and the whole EmERGE Consortium. NVivo 11 software (QSR International Pty Ltd) was used to support the management of the textual data and to organize the codes being assigned to the transcripts.

**Table 1:** Data collection across study sites.

Study site and mode of data collection		Participants’ characteristics					
		Male		Female		Total participants	
		PLWH^a^	Clinicians	PLWH	Clinicians	PLWH	Clinicians
**Brighton (Br)**							
	1 workshop PLWH	7	—	1	—	8	—
	1 workshop PLWH	6	—	3	—	9	—
	2 interviews PLWH	1	—	1	—	2	—
	1 workshop clinicians	—	3	—	8	—	11
	1 interview clinicians	—	—	—	1	—	1
**Lisbon (Li)**							
	1 workshop PLWH	4	—	4	—	8	—
	1 workshop PLWH	7	—	3	—	10	—
	4 interviews PLWH	4	—	—	—	4	—
	1 workshop clinicians	—	2	—	11	—	13
	1 interview clinicians	—	1	—	—	—	1
**Antwerp (An)**							
	1 workshop PLWH	9	—	1	—	10	—
	1 workshop (mixed)	5	1	1	2	6	3
	3 interviews PLWH	3	—	—	—	3	—
	1 workshop Clinicians	—	4	—	9	—	13
**Zagreb (Za)**							
	1 workshop PLWH	5	—	2	—	7	—
	1 workshop (mixed)	3	2	—	2	3	4
	5 interviews PLWH	4	—	1	—	5	—
**Barcelona (Ba)**							
	1 workshop PLWH	9	—	1	—	10	—
	1 workshop (mixed)	5	1	1	4	6	5
	6 interviews PLWH	6	—	—	—	6	—
	1 workshop clinicians	—	5	—	7	—	12
Total (7 workshops PLWH; 3 workshops [mixed]; 20 interviews PLWH; 4 workshops clinicians; and 2 interviews clinicians)		78	19	19	44	97	63

^a^PLWH: people living with HIV.

### Design Specification and Priorities

A further step in co-design involved ensuring that the outcomes of workshops and interviews were embedded in decision making. Therefore, the outcomes of our study were presented and discussed at EmERGE Consortium meetings, where lead clinicians from each study site, PLWH (represented through EATG), researchers (from University of Brighton), and technology developers were represented and had the opportunity to provide feedback. Following these initial discussions, a technical co-design group, consisting of 2 representatives from these parties (clinicians, PLWH, researchers, and developers), was formed to outline a design specification document and discuss priorities for the development of functionalities and features to be included in the prototype platform. This group held several meetings and worked in partnership with community partners, clinicians, and the project consortium on an ongoing basis to ensure that priorities were reviewed and rearticulated in light of ongoing changes and specific contexts. In the following section, we present the results from co-design workshops and interviews. Thereby, we use quotes from participants, only indicating participant (P=PLWH; C=clinician), study site (by first 2 letters), and mode of data collection (WSm=workshop mixed, I=interview), for example, P_Za_WSm. In the discussion section of the paper, we will outline how these results informed the development of the EmERGE prototype platform.

## Results

### Overview

As Textbox 1 illustrates, we established our findings into 3 broad thematic clusters: (1) *approaching the mHealth platform*, (2) *imagining the mHealth platform*, and (3) *anticipating the mHealth platform’s implications*. At the start of the co-design workshops and interviews, we introduced the aim of the co-design study, namely, to explore the concept of an mHealth platform to participants. One specific aim of our study was to elicit experiences of living with HIV or working in HIV care. Therefore, before discussing the possible functionalities that could be included within such a platform, we asked participants to broadly reflect on their experiences of living with HIV or of working as an HIV clinician. Throughout the data analysis, it became evident that these general experiences formed the ways in which PLWH and clinicians were reflecting upon possible functionalities, opportunities, and drawbacks of an mHealth platform. To indicate this, we labeled initial themes as questions by which PLWH and clinicians were *approaching the mHealth platform*. From the backdrop of these approaches, our participants started *imagining the mHealth platform* and articulated tentative interpretations about what an mHealth platform for HIV care could *do*. Thereby, linked to our second study aim, potential functionalities and components of the platform were discussed. Once potential functionalities were conceptualized, study participants then elaborated connections between technology functions and the wider context within which it will be utilized. Thereby, participants anticipated the platform’s potential implications for self-management and the provision of health care and, thus, contributed to our third study aim to understand the potential benefits of, and concerns about, mHealth.

### Approaching the mHealth Platform

Both PLWH and clinicians approached discussions about the proposed mHealth platform with pre-existing concerns arising from their experiences of receiving or providing HIV care. While broadly reflecting on experiences of living with HIV, PLWH particularly addressed the issue of stigma and the ways in which they attempt to take control of HIV. Clinicians working in the field of HIV care focused on their experiences with digital technologies and questioned what type of patient will be capable of engaging with mHealth.

#### Patients’ Approaches

##### Renegotiating Stigma?

Experiences of stigma were a topic within most of the workshops and interviews. Several PLWH argued that the general public as well as some health care professionals still lack knowledge about HIV. To illustrate this impression, 1 participant reported an incident where his neighbor, while inviting him for coffee, told him: “...but bring your own mug” (P_Za_WS). Other participants referred to situations where they have been suspended from work when their employer found out that they were HIV positive. Due to such experiences, many participants stated that they do not disclose their HIV status to friends or even their families and thus often feel isolated as they “don’t have someone to talk about [HIV]” (P_Za_WSm, male). Moreover, when engaging with health care professionals, some PLWH are reluctant to disclose their HIV status:

So even when you go for medical check-ups, you keep quiet...you don’t want to be put in that situation where you’re labelled, and where you wouldn’t get the best possible medical care just because you are HIV positive.

P_Za_I, male

Due to such experiences, PLWH approached the mHealth platform by questioning how it would *renegotiate stigma*. Thereby, the platform’s functionalities and features were discussed against the backdrop of protecting or jeopardizing confidential HIV data (see section Security and Privacy), and suggestions were made to use the platform to inform the broader public to reduce the stigma of HIV (see the section Changing Public Attitudes Toward HIV).

##### New Opportunities for Control?

The heterogeneous practices of keeping in control of one’s condition were an integral part of patients’ illness narratives. Thereby, the adherence to the antiretroviral medication regime was a pressing element: “I know if I don’t take the pills I die” (P_Br_WS, male). Most PLWH argued that they use alarms to remember taking their medicines: “with my mobile phone, I set the time...to take medication. For otherwise I forget” (P_An_I, male). Some PLWH, however, stated that they manage their medication intake by integrating it within their “natural schedule” (P_Li_I, male) of everyday routines and thus do not rely on additional reminders.

**Thematic clusters and categories.**

- Approaching the mHealth platform (experiences)
  - Patients’ approaches: Renegotiating stigma? New opportunities for control?
  - Clinicians’ approaches: Compatibility and added value? Who constitutes the target group?
- Imagining the mHealth platform (functionalities)
  - Medical functionalities: accessing test results; managing medicines; managing appointments; and digital communication channels
  - Social functionalities: peer support network; international travel; and changing public attitudes toward HIV
  - General features: security and privacy; credibility; language; sensitivity for disabilities; costs; training and tutorials; and other technicalities
- Anticipating the mHealth platform’s implications (benefits and concerns)
  - Implications for self-management: Creating (un)certainty? Reconfiguring relationships? Altering the understanding of health?
  - Implications for health care provision: Replacing traditional care pathways? Rationalities of mHealth? Effects on workload?

Dealing with situations of uncertainty was another important aspect of managing HIV. Such situations can occur when pain or certain symptoms are experienced by PLWH, but they are unsure if these are side effects of HIV medication or part of the normal process of “getting older” (P_Li_WS, female). To deal with such situations, PLWH have to seek for information and advice. Although the HIV consultant was seen as the most trusted source of health information, several PLWH argued that general practitioners (GPs) are often not capable of providing adequate advice as they often lack basic knowledge about HIV and thus are inclined to relate every medical problem to HIV: “you go to the GP, he will tell you ‘It’s HIV’” (P_Br_WS, female). Community groups were perceived as another important source of health information. For specific health-related problems (eg, side effects of medication), PLWH valued expertise based on experiences from someone who has *lived through it*. With these concerns in mind, PLWH approached the mHealth platform by questioning how it could create new opportunities for control. For example, they asked whether the platform could be seen as a device to keep on top of one’s treatment adherence (see the section Managing Medicines and Managing Appointments) and if it could offer new ways to get in touch with health care providers (see the section Digital Communication Channels) or community groups (see the section Peer Support Network).

#### Clinicians’ Approaches

##### Compatibility and Added Value?

Clinicians often discussed their hospital’s technological systems and reflected on whether the mHealth platform would be compatible and provides added value. Some clinicians expressed a general impression that the health care sector is slow in adopting new technologies, saying, “...in the NHS we fall way behind the rest of the world in terms of using social media and electronics in managing our patients” (C_Br_WS, male doctor).

This was seen as problematic because by considering technological advances, the limits of *old* technological systems became apparent. In this way, a clinician in Zagreb illustrated the limits of discussing blood test results through telephone lines:

...we ask people [patients living remote from the clinic] to call in approximately two to three weeks after they have been to the visit [where blood was drawn] so that we can discuss the new lab result and I don’t think that the system is working perfectly because we cannot answer the phone every time…it happens that the patient doesn't know his lab result until his next appointment.

C_Za_WSm, male doctor

In similar ways, the limits of existing email communication with patients (Brighton) with respect to data security were addressed or an existing telecommunication platform for virtual video consultations (Barcelona) was criticized for being only accessible through a conventional Web interface (and not through a smartphone app). Established technological systems, however, were also valued because they have already been adopted within work practices. Therefore, some clinicians questioned if the proposed mHealth platform could be integrated into established systems:

I think that [the mHealth platform] is useful if we have one instrument...if it’s just one more instrument it’s not worth it ’cos it’s time-consuming, so I think it it’s important to be a platform that connects with the existing ones.

C_Li_WS, female clinician

From the backdrop of their work routines and experiences with established technological systems, clinicians approach the mHealth platform by questioning its added value and compatibility. In this regard, clinicians asked how the integration of the platform would affect their workloads (see the section Effects on Workload).

##### Who Constitutes the Target Group?

Clinicians also addressed the diversity of the patient population. Several clinicians emphasized how important it is “to identify very carefully what kind of patient can improve with this [platform]” (C_Ba_WS, male doctor). In terms of patients’ health conditions, most clinicians argued that only patients that “have been doing well for many years” (C_Li_WS, female doctor) and are medically “stable” (C_An_WS, female clinician) should be considered as target group for the mHealth platform. Stable HIV patients have controlled viral loads and cluster of differentiation 4 (CD4) counts, and this was seen as essential in relation to the platform’s functionality *accessing blood results* (see below). It was outlined that, for stable patients, the blood test is rather a routine checkup, and thus, the results are hardly surprising because they are mostly within a certain range. These patients, therefore, would—presumably—not become unsettled when retrieving lab results on a smartphone app. In the case of nonstable patients, however, results are fluctuating, and several clinicians emphasized that such patients would not be able to interpret and make sense of these fluctuations outside of a face-to-face consultation. In a clinical consultation, the meaning of the result can be assessed and implications for the treatment negotiated from the backdrop of patients’ broader psychosocial health issues. Hence, it was argued that nonstable patients should not have access to their results before a clinical consultation. Furthermore, it was emphasized that patients require “a certain skill set” (C_Br_I, female nurse) to use the smartphone app (provided with the mHealth platform) and to interpret data and information that are made available on it. Therefore, most clinicians considered only “highly educated” (C_Li_WS, female clinician) and “experienced” (not newly diagnosed) patients (C_Ba_WS, female clinician) as potential target group for the mHealth platform (see the section Creating (Un)certainty?). These approaches had a significant impact on how clinicians discussed potential functionalities and implications of mHealth. This will be outlined in the following section.

### Imagining the mHealth Platform

From the backdrop of the experiences of receiving or providing care, participants imagined and articulated potential functionalities and features that could be provided through mHealth. Although clinicians largely suggested functions that we conceptualized as *medical functionalities*, PLWH, additionally, considered *social functionalities* and *general features* in more depth.

#### Medical Functionalities

##### Accessing Test Results

Co-design participants discussed whether the mHealth platform, or rather *the app* provided for use by patients through this platform, could provide access to blood test results:

If I could receive...the results of my blood tests through the app, I wouldn’t have to go to the internist...the app could save me a whole lot of health expenses.

P_An_WS, male

In line with this statement, several PLWH and clinicians argued that for some patients (see the section Who Constitutes The Target Group?), regular appointments are just an exertive routine to collect blood results. By having remote access to results, it was argued that some routine visits to the clinic could be avoided, and this would contribute to patients’ quality of life (by saving traveling and waiting time and/or avoiding the potential disclosure of the HIV status by encountering people in the clinics’ waiting rooms). Furthermore, it was emphasized that by having access to medical data, patients can become more knowledgeable about their condition (see the section Altering the Understanding of Health?) and could share data with health professionals to agree on treatment decisions: “the analysis could be showed to other doctors” (P_Li_WS, male). However, questions were raised about how patients could best be enabled to interpret results. Most clinicians suggested that only “a selected part of the blood analysis” (C_Ba_WS, male doctor) should be sent to the patient. There was a certain consensus among clinicians that, rather than sending the whole analysis, results should be restricted to viral load, CD4 count, cholesterol levels, and kidney and liver function. Furthermore, it was emphasized that results could be accompanied with a “small message” (C_An_WS, female clinician) from the doctor or a “sort of colour coding” (P_Br_WS, male) that helps to interpret the results.

##### Managing Medicines

As described above, patients approached the mHealth platform with concerns about maintaining control over their health. The adherence to the medication regime, in particular, was perceived as a considerable stress factor among several PLWH. These concerns were reflected in how they *imagined* the platform and its functionalities. PLWH and clinicians mentioned that a tool to assist the management of medicines would be an important function in the app. In particular, such a function would include reminders to take medicines. Although several PLWH stated that they already set reminders on their smartphones, it was argued that an app, provided by the mHealth platform, could offer a more comprehensive reminder system. In this regard, PLWH referred to complex social situations where they were not immediately able to take their medication when the alarm on the smartphone goes off and thus were unsure at a later point whether they had actually taken their medication. Therefore, PLWH discussed the possibility of a “snooze” button (P_Br_WS) and additional reminders that “will just keep reminding you until you do it [take the medicine]” (P_Za_I). Another problem was drug interactions, as 1 participant pointed out:

[Doctors] give me medication that interfered with my retroviral drugs...there should be something that we ourselves could manage this situation...An application where we could see if we can take the drugs A, B or C with this cocktail of antiretroviral drugs.

P_Li_WS, female

In line with this quotation, several PLWH stressed that health care professionals (aside from the HIV consultant) often lack knowledge about the interactions of the medicines. Therefore, an option to recheck interactions within an mHealth platform was seen as essential.

##### Managing Appointments

Some PLWH reported that they quite often miss appointments for their medical check-ups:

I have missed a lot of appointments. I think it would be useful to have some reminders to tell me.

P_Li_WS, female

Accordingly, both PLWH and clinicians regarded reminders as an important tool to ensure “that people come to their appointments” (C_An_WS, female clinician). Moreover, the booking of appointments was experienced as stressful by some PLWH, who said, “...many times I’ve had to change my appointments, and I’m ringing up and I can’t get through.” (P_Br_WS, female).

Both arranging appointments through the telephone and at the reception desk were experienced as time consuming. Therefore, PLWH stated that the mHealth platform could offer “an online calendar, if there is an empty spot that suits you...you click on it” (P_Ba_WS, male).

##### Digital Communication Channels

Participants explored the different ways in which the mHealth platform might offer communication channels between PLWH and health care providers. One option, particularly emphasized by clinicians, was indicated as “push notifications” (C_Br_WS, female clinician). This was seen as a way for clinicians to inform specific patients about new trials, medical innovations, or ways to maintain a healthy lifestyle. Some PLWH emphasized that they would appreciate a “newsfeed” (P_Ba_WS, male) that notifies “if something new comes out in the world of HIV inventions” (P_An_WS, female) or provides information about “diets, sports, sex, whatever!” (P_Ba_WSm, male). However, several PLWH proposed a “direct messaging service” (P_Ba_WS, male) where messages could be exchanged in both ways—between patients and clinicians; this would help in situations of uncertainty where medical advice is required. Among clinicians, however, two-way communication messaging was seen as more controversial. Although some clinicians regarded it as essential that patients could contact their clinician in case of problems or questions, others had “misgivings about having a two-way communication...because we’re fighting the wolf...volumes of free text” (C_Br_WS, male doctor). The quotation illustrates that some clinicians feared that a two-way messaging system would produce an unmanageable workload (see the section Effects on Workload?). Another option for communication that participants discussed was *virtual consultations*. Although video consultations seem to most appropriately simulate the face-to-face consultation, they are still embedded within potentially constraining spatiotemporal contexts. As 1 participant puts it:

I probably wouldn’t use video calling, because...you have to schedule in a time, make sure you’re in a specific place that can be completely private to have that conversation.

P_Br_I, male

#### Social Functionalities

##### Peer Support Network

While reflecting upon functionalities of the mHealth platform, the distinction between medical and social aspects was emphasized by PLWH:

I do see a difference between the medical world and the rest of the world...nearly all information comes from doctors...there is no community anymore...managing, eh, your infection is also about sharing your experience.

P_An_WSm, male

To complement medical knowledge with experiential expertise, some PLWH argued that the platform should facilitate a peer support network through a chat function. Besides the exchange of experiences, a chat forum was also seen as an opportunity for PLWH to overcome isolation. In this way, 1 participant argued that:

[T]hey [clinicians] are not here at midnight...when you are scared and wake up in tears and shaking: “What do I do, oh my God, I’m HIV positive.” So I think that it will be a very good thing that you can go on the app and see someone online and only talk about HIV or about the weather...

P_Za_WSm, male

##### International Travel

PLWH mentioned international travel as another aspect of social life that could be supported by the new mHealth platform. It was suggested that it could provide general information about the implications of traveling with HIV, such as travel restrictions to certain countries, information about travel documents (eg, certificates for the antiretroviral medication), and advice for situations of emergency (eg, losing the medications). Furthermore, PLWH discussed whether an mHealth platform could help to manage medicines between different time zones, saying, “...it’s important to address the issue of schedules of intakes of the medicines when traveling and changing time zones” (P_Li_I, male).

##### Changing Public Attitudes Toward HIV

In regard to the target group of the mHealth platform, some PLWH stated that it should not only be for “us [PLWH], but for everyone” (P_Ba_WS, male), as by providing information on HIV to the general population, the platform could “take away a bit of the discriminatory burden of this disease” (P_Li_WS, male).

#### General Features

##### Security and Privacy

As a general feature, the mHealth platform (and its related app) needs to provide “some other standard of security” (C_Ba_WS, male doctor). This quotation illustrates the perspective of most study participants (PLWH and clinicians) who emphasized that because of the high stigma around HIV, the platform should accomplish the highest level of security and privacy. In this way, participants argued that the app design should be discrete—“it should not have 1000 red ribbons” (P_An_WSm, male)—and posed questions such as how the medical records would be encrypted, how the data would be stored, and by whom it would be managed or shared. Although there were several questions with regard to security and privacy, most PLWH stated that they already use apps for banking and other purposes and thus would trust an mHealth platform if it accomplishes a similar level of security. Some other participants, however, rejected the idea of having their confidential HIV data processed through an mHealth platform:

Even though it has codes and all kind of stuff…this app is online…Anybody can hack my email…I don’t want it to maybe one day, come out…if you say, “This app is here you can download it,”I will say:“No, thank you.”

P_Za_I

In discussing these security concerns with the EATG and our community partners, it was elaborated that there is a central distinction between banking apps and HIV health apps. The point was made that there is a general expectation that banks will return any money lost to you through security failure on their part. Money can be paid back, but the disclosure of sensitive health information cannot be undisclosed and may have a significant impact on people’s lives.

##### Credibility

Credibility was another topic that emerged in our data. PLWH often stated that it is important that the mHealth platform and its related app come from a trusted institutional body. PLWH pointed out that, for them, health care providers and patient organization have more credibility than pharmaceutical companies.

##### Language

The language used by the mHealth platform was another discussion point among PLWH who underlined that it should be available in their local language and the language should not be “over-complicated” (P_Br_WS, male).

##### Sensitivity for Disabilities

Sensitivity for disabilities was an issue mentioned by some participants. In particular, an option for voice recognition and a text narrator built within the mHealth platform was considered as important to make the app accessible for users with less eyesight, dyslexia, or for people who are analphabetic.

##### Costs

The app provided by the mHealth platform, according to most PLWH, should be free of cost. However, some PLWH expressed willingness to pay for a good-quality app, saying, “...it would not necessarily have to be completely free of charge, because you don’t get something for nothing, but maybe the basic version could be free” (P_An_WS, male).

##### Training and Tutorials

The importance of some kind of training to use the mHealth platform and its app was stressed by both clinicians and PLWH. It was argued that PLWH could be introduced to the app by health care professionals, within specific training workshops or through tutorials that are included within the app.

##### Other Technicalities

PLWH addressed a range of other technicalities that they perceived as relevant for an app. Thereby, the app’s battery and memory consumption within the smartphone as well as its speed were questioned. An offline access to the information within the app was considered useful. Moreover, it was suggested that an option to individualize the app would be important. Options to individualize the app were seen in selecting the functionalities one wants to see on the app’s dashboard, choosing one’s own app icon, or selecting different types of reminders (eg, for medication intake).

### Anticipating the mHealth Platform’s Implications

Co-design participants also discussed the potential implications of an mHealth platform for self-management and for the provision of health care. These implications were debated quite controversially, emphasizing both benefits and risks. To indicate these controversies, we labeled the following themes as questions.

#### Implication for Self-Management

##### Creating (Un)certainty?

Both PLWH and clinicians debated whether an mHealth platform would contribute toward certainty or uncertainty within the self-management of HIV. Some participants were convinced that receiving results through an mHealth platform would create more certainty and reassurance. In this way, 1 participant explicated:

To access something, that seems quite interesting, you know, and perhaps just to check the percentage of your CD4 and your viral loads...that’s all about reassuring and taking care of your health condition, even just doing those quick checks. But you’re in control of it.

P_Br_I, female

In addition, some clinicians were convinced that experienced patients would be able to interpret blood test results, acknowledging that “patients have learnt quite quickly to speak the language of HIV bloods” (C_Br_I, female nurse). It was argued that through an mHealth platform, PLWH could become more informed and reassured. These positive perspectives toward the platform were rejected by other participants who pointed out that having instant access to medical results outside of a face-to-face clinical encounter could create anxiety and uncertainty among PLWH.

...anxiety about your results, because you don’t know how to interpret them, you may have a blip on your viral load and that means nothing, but if you have access you may be anxious for a couple of days before going to the doctor, and she explains to you that’s nothing.

P_Li_I, male

From the perspective of these participants, medical results are best discussed within a face-to-face clinical encounter. Thereby, assistive measures that could help to interpret the results accessible through the platform (eg, color coding, see the section Accessing Test Results) are not seen as sufficient to inform health decisions. Such decisions, according to these participants, are best embedded in the physical clinical encounter where individual feelings and illness experiences can be expressed and treatment decisions can become the product of a deliberative process of care, balancing experiential and medical knowledge.

##### Reconfiguring Relationships?

Another discussion emerged around the question of how mHealth would affect the relationship between patients and clinicians. Most PLWH mentioned that they have built a strong relationship with their HIV consultant and were “loyal” (P_Li_I, male) to them over a long period. In this regard, some were worried that, if communication would move from the physical encounter toward mHealth, “the relationship [with the consultant] is not the same” (P_Ba_I, male). Furthermore, clinicians emphasized that it is crucial to first establish a relationship with patients in face-to-face interactions but emphasized that this could then be moved to digital communication. In this way, a female doctor suggested to PLWH in a mixed workshop:

once...we already have a previous relationship, we already know each other...I want to propose to you to stop seeing you in face-to-face visits.

C_Ba_WSm

Some PLWH stressed the potential of the mHealth platform and the related app to create a closer relationship with clinicians, suggesting “an app that you can use to be in touch with your own doctors” (P_An_I, male). To do this, as was acknowledged, the platform would need to provide two-way communication (see the section Digital Communication Channels).

##### Altering the Understanding of Health?

Another point discussed among PLWH was how far the mHealth platform could contribute toward a more pronounced understanding of one’s HIV condition. Most PLWH saw data as essential to gain knowledge about one’s condition. In this way, the potential of the platform to store the medical history and visualize it on images or graphs was outlined. This was valued for enabling an in-depth understanding of the body and new options to monitor and control HIV:

Regarding the history, it’s always important. I think that it’s through there that we can reach conclusions about what is doing us better, doing us worst and maybe one day to see a marker and realize “Look, after all THIS is what degraded THIS.”

P_Li_WS, female

...it might be nice to have something that’s sort of telling you, you know, where you are on the scale.

P_Br_WS, male

Other PLWH, however, argued that to become knowledgeable about their health, they privilege their own (bodily) experience, feelings, and self-awareness:

I know myself and I know when I don’t feel good...Sometimes it’s better to ignore some things like that [medical data]. I mean you are living with the disease, but you don’t want to think about it every day.

P_Za_I, female

These participants emphasized that through an mHealth platform that pushes health data and alerts, they would be constantly confronted with their disease. Technology was thus perceived as invasive, disrupting practices of everyday life, and exposing PLWH to the risk of becoming “obsessed” (P_Ba_I, male) with their condition.

#### Implications for Health Care Provision

##### Replacing Traditional Care Pathways?

Participants questioned whether an mHealth platform would replace or complement routine face-to-face consultations within the traditional pathway of HIV care. In this respect, both clinicians and PLWH agreed that some face-to-face clinical encounters are essential for effective HIV care. As the following quote illustrates, some participants argued that social aspects such as sexual practices, relationships, and family problems could not be appropriately discussed through digital communication channels:

I don’t see myself sitting behind a computer and having a discussion about relationships...for me that's a drawback.

C_An_WS, male doctor

Furthermore, it was stressed that only a personal relationship with the patient would facilitate a good consultation around social issues. Some clinicians also emphasized that:

We do a physical exam and patients sometimes are not aware of...physical appearance or the presence of symptoms.

C_Li_WS, female clinician

An appropriate physical examination, it was argued, could not occur outside the face-to-face encounter. By pointing to such restrictions, both clinicians and PLWH emphasized that an mHealth platform should complement, rather than substitute, face-to-face visits:

I don’t think that this kind of...application should be a substitute of the medical visit...But, they can work together.

P_Li_I, male

##### Rationalities of mHealth?

Other concerns were related to the rationalities behind the utilization of an mHealth platform. In particular, it was questioned whether such a platform would be utilized to improve the quality of care or to downsize health care expenditure. Some clinicians pointed out that an mHealth platform would probably save resources but, at the same time, could contribute in “improving convenience and facilitating the access [to healthcare]” (C_Ba_WS, male doctor). However, some PLWH expressed worries that the platform “was intended to make savings in healthcare and decrease the number of visiting hours at the doctors’ [office]” (P_An_WSm).

##### Effects on Workload?

A discussion point among several clinicians was whether the mHealth platform would increase or decrease their workload. In this way, 1 clinician stated:

I’m interested in how this [the mHealth platform] would work around our end and to see how much...drudgery can be taken out of the work...‘cos...there's a lot of work processing records.

C_Br_WS, male doctor

Although some participants were convinced that the platform could save time and clinicians “would have more resources for other things” (C_Ba_WSm, female doctor), others argued that it would require more work:

...for a clinician if you have to do that, you have to go to the results, interpret the results and then loading them onto the website or the app or the platform, and then there’s also additional, yeah, workload.

C_An_WS, male doctor

## Discussion

### Implications and Comparison With Prior Work

Participation is considered a key principle for designing health interventions and technologies in ways that are accessible and meaningful to people in different life situations [23,29,30]. In this section, we compare the outcomes of our co-design process with the results of previous studies that involved PLWH in the design of HIV apps and highlight how co-design findings can inform mHealth developments. We detail this by illustrating how the findings informed the development of the EmERGE platform.

#### Target Group for HIV mHealth

The complexities of interpreting the meanings of viral load and other numerical definitions of HIV health have been widely discussed in relation to self-managing HIV [31,32]. Our results reflect this literature by highlighting patients’ and clinicians’ concerns that having access to one’s health data requires the ability to interpret and use these data. These capacities were not anticipated to be equally distributed among population groups. In the context of HIV care, we found that years since diagnosis with HIV and the relative stability of the HIV condition can configure the meaning of having access to blood test results and other quantified health data [22]. In the case of newly diagnosed and unstable HIV conditions, direct access to numbers was associated with bringing anxiety and uncertainty into care practices. In the study of Swendeman et al [18], clinicians suggested several ways in which mHealth devices should particularly address newly diagnosed patients and patients with comorbidities. Our data support the view that medication reminders, options to monitor blood results, etc, are particularly useful for these patient populations. However, our analysis suggests that when implementing mHealth with newly diagnosed or unstable patients, the most recent test results should be discussed within the context of a face-to-face clinical encounter before being sent to a patient’s mobile devices.

#### Medical Functionalities

In current HIV medicine, there is a strong focus on viral suppression as the ultimate goal of ART. Therefore, both the collection of biomarkers by means of blood tests and the close surveillance of patients’ adherence to the treatment regime play a key role in monitoring HIV progression [33]. This is reflected in our findings as well as those of other studies where the storing and tracking of lab results and medication and appointment reminders were identified as desirable app functionalities for PLWH [14-18]. Access to blood test results has been imagined and anticipated as being an important function by both patients and clinicians. Clinicians in the study of Swendeman et al [18] additionally pointed out that messages or feedback from health care providers to patients could be used to enhance patient motivation to treatment adherence. Although two-way communication features have not yet been considered within the development of comprehensive HIV mHealth platforms based on interacting apps and Web interfaces [14-16], with options to exchange messages between patients and clinics currently being implemented through basic cell phone text messaging (short message service) services [34], many of our co-design participants did regard this as an important functionality to maintain relationships and exchange information and concerns with their care providers. However, although the inclusion of two-way communication functions might be considered as an important direction for the development of mHealth, there remain significant challenges concerning how to achieve this in practice. In the case of EmERGE, discussions about how two-way communication between clinicians and patients could be facilitated in a way that does not significantly increase clinicians’ workloads are still ongoing, and this feature is not yet realized within the new care platform.

#### Social Functionalities

PLWH imagined several social functionalities that would support them in managing their HIV condition. Continued exchange among peers is important for individuals to feel less alone while engaging in chronic illness self-management and to generate knowledge that is based on personal experiences [35]. However, frequent interaction with peers may often not be feasible because of spatiotemporal limitations or fears of stigmatization in face-to-face environments. Along with other studies, our findings underscore perceptions of potential users that mHealth could facilitate a comfortable and safe environment for PLWH to engage in peer support [14-17,36]. In addition, our participants imagined an app supporting them while traveling internationally (eg, managing HIV intake across time zones) or help to make the general public more knowledgeable about HIV. At the time of writing, this has not been included in the first iteration of the platform but remains an option for later developments.

#### General Features

Our findings have highlighted that both patients and clinicians anticipated it as essential that an mHealth platform for HIV care should be based on the highest standards of security measures. Concerns about security and privacy implications of mHealth for HIV have also been stressed by previous studies [14-16]. However, our study (as well as one other [18]) has also revealed clinicians’ perspectives that novel mHealth platforms could provide higher standards of security as compared with older technologies such as email communication. Many other general features (such as credibility, news feeds, simplicity, cost, and customizability) have been identified as important through our research as well as previous studies [15,17] and should be considered in mHealth developments. Working in a multilingual European context, we found that it was of great importance that mHealth apps are available in the local language. In the case of EmERGE, the app will offer the option to choose between the 5 main languages spoken in our study sites.

#### Implications for Further Research

In the field of digital health studies, it has been stressed that many of the social and ethical consequences of mHealth remain under-researched [37]. Through the first stage of a co-design process, developed and implemented as a central component of the EmERGE project, participants had the opportunity to express their concerns and anxieties about mHealth, and our analysis of the results of this process has enabled us to help fill this gap in the literature. This paper has illustrated how working closely with clinicians and PLWH in a process of co-design can contribute to a fuller understanding not only of the perceived benefits associated with mHealth but also of the potential unintended or negative consequences that users envisage and how these insights can be reflected in mHealth platform design. However, this is just the first stage of the co-design work in EmERGE. Our approach to co-design is set within a broad sociotechnical understanding of digital health developments that recognizes design as a continuous and co-constituting process that begins before the technology itself is present and continues well into implementation and use phases. Once in use, platforms, apps, and websites are in a continuous process of transformation [38]. They require constant *fixes*, *updates*, and *versions*, not only because of technological changes but also because of necessary sociocultural developments that accompany them. Co-design thus requires ongoing engagement with actual practices where technology has to be tamed and tinkered with to fit specific situations of usage [39]. Engaging with practices of design and use provide valuable insights into how people approach, imagine, anticipate, and ultimately interact with technologies, and this can contribute toward an understanding of the situations and conditions within which mHealth can facilitate or undermine practices of care [22,40]. We propose that co-design approaches that are continuous throughout the lifecycle of mHealth interventions are likely to provide timely and relevant insights toward the creation of meaningful and effective mHealth solutions. As the EmERGE mHealth platform is fully integrated into the local care pathways, we will continue this co-design work with clinicians and PLWH as we seek to investigate and to improve the technology *in-use* in specific contexts.

### Limitations

Although we aimed to recruit a broad variety of co-design participants, among PLWH, white gay men were over-represented compared with women and migrant groups. Among clinicians, female doctors were over-represented in comparison with male participants and clinicians from other medical backgrounds (nurses, psychologists, etc). We also have to assume that participants that were more likely to be interested in the use of mobile technology agreed to participate in this study. These recruiting issues restrict the potentialities to generalize the outcomes of this study. Another constraint might be intrinsic to technology development projects. The limited time frame between data collection and the start of the development of the mHealth platform meant that the actual coding of the data could only be performed by 1 researcher. However, to enhance the rigor of the data analysis, initial codes and categories were discussed and negotiated between the research team on a bimonthly basis. Confirmation of reliability was also provided through feedback from community partners and the whole project consortium gathered at various meetings. Although findings presented in this paper have highlighted patients’ and clinicians’ *perspectives* toward desirable, or hypothetical, functionalities for HIV mHealth apps, the next phases of our project will enable us to evaluate the actual impact of these functionalities on health *experiences* and *practices*.

### Conclusions

In this study, participatory co-design methods have been used to (1) elicit experiences of living with HIV and of working in HIV care, (2) identify functionalities and features for an mHealth platform that PLWH and clinicians regard as useful for HIV treatment and care, and (3) identify potential benefits as well as risks and concerns of such a platform. Through our analysis, we have highlighted how this process allowed us to better understand how clinicians and patients were approaching, imagining, and anticipating what it is that the platform could *do* for HIV care. Our approach to co-design enabled us to facilitate early engagement in the mHealth platform, enabling patient and clinician feedback to become embedded in the development process at a preprototype phase. Although the technologies in question were not yet present, understanding how users approach, imagine, and anticipate technology formed an important source of knowledge and proved highly significant within the technology design and development process. At the time of writing, the platform has been implemented and is being more fully evaluated in 5 clinical sites in the context of the wider EmERGE study. Co-design work will continue as users’ experiences of the new mHealth-based care pathway are captured and shared both within and between sites to inform further developments of the EmERGE HIV platform. Future papers will explore these later phases of co-design and draw out the implications of our approach and findings for mHealth developments in HIV care.

## Abbreviations

ART: antiretroviral therapy
CD4: cluster of differentiation 4
EATG: European Aids Treatment Group
EmERGE: Evaluating mHealth Technology in HIV to Improve Empowerment and Health Care Utilization: Research and Innovation to Generate Evidence for Personalized Care
EU: European Union
GP: general practitioner
NHS: National Health Services
PLWH: people living with HIV
